# NPM3 as a novel oncogenic factor and poor prognostic marker contributes to cell proliferation and migration in lung adenocarcinoma

**DOI:** 10.1186/s41065-023-00289-6

**Published:** 2023-05-31

**Authors:** Shan Wei, Jing Xing, Kaining Lu, Kai Wang, Wanjun Yu

**Affiliations:** 1 Department of Respiratory and Critical Care Medicine, The Fourth Affiliated Hospital, School of Medicine, Zhejiang University, Yiwu, 322000 Zhejiang People’s Republic of China; 2 Department of Respiratory and Critical Care Medicine, The Affiliated People’s Hospital of Ningbo University (Ningbo Yinzhou People’s Hospital), No.251, Baizhang Road, Ningbo, 315040 Zhejiang People’s Republic of China; 3 Ningbo University School of Medicine, Zhejiang Province, Ningbo, People’s Republic of China; 4 Department of Urology, The Affiliated First Hospital of Ningbo University (Ningbo First Hospital), No.59, Liuting Street, Ningbo, 315010 Zhejiang People’s Republic of China

**Keywords:** Lung adenocarcinoma, NPM3, Prognosis, Immune microenvironment, Cell proliferation, Cell migration

## Abstract

**Background:**

Lung cancer is the leading cause of cancer-related deaths worldwide, and despite recent advances in targeted therapies and immunotherapies, the clinical benefit remains limited. Therefore, there is an urgent need to further investigate the molecular mechanisms underlying lung cancer. The aim of this study was to investigate the expression and function of NPM3 in the tumor microenvironment of lung adenocarcinoma (LUAD).

**Methods:**

We utilized bioinformatics tools and databases, including UALCAN, GEPIA2, HPA, and Sangerbox, to analyze NPM3 expression in LUAD samples and its association with prognosis and mutational landscape. NPM3 expression in various cell types was assessed at the single cell level using the TISCH database. We also used algorithms such as TIMER and EPIC to explore the crosstalk between NPM3 expression and immune features. KEGG enrichment analysis was performed to identify potential signaling pathways of NPM3. Finally, we employed siRNA knockdown strategy to investigate the effect of NPM3 on LUAD cell proliferation and migration in vitro.

**Results:**

NPM3 was significantly upregulated in LUAD tissues and was strongly associated with poor prognosis and *TP53* gene mutations. Single-cell sequencing analysis revealed that NPM3 was expressed in immune cells (dendritic cells and monocytes/macrophages) in the tumor microenvironment. Moreover, NPM3 expression was negatively associated with immune B cell and CD4 T cell infiltration, as well as with several immune-related genes (including CCL22, CXCR2, CX3CR1, CCR6, HLA-DOA, HLA-DQA2). KEGG enrichment analysis indicated that NPM3 expression was associated with cell cycle, CAMs, and NSCLC pathway genes. Finally, in vitro experiments showed that NPM3 knockdown inhibited LUAD cell proliferation and migration in NCI-H1299 and SPC-A1 cells, and suppressed the expression of CCNA2 and MAD2L1.

**Conclusion:**

Elevated NPM3 expression predicts poor clinical outcome and an immunosuppressive microenvironment in LUAD tissues. NPM3 promotes LUAD progression by promoting cell proliferation and migration, and targeting NPM3 may represent a novel therapeutic strategy for LUAD.

**Graphical Abstract:**

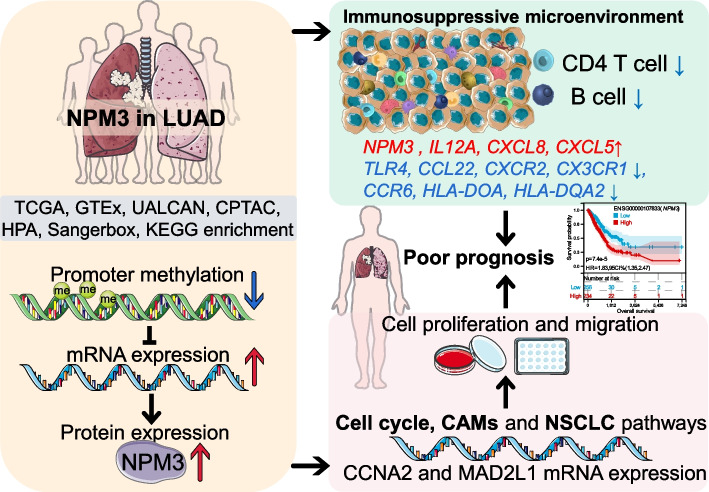

**Supplementary Information:**

The online version contains supplementary material available at 10.1186/s41065-023-00289-6.

## Introduction

Lung cancer is the leading cause of cancer mortality worldwide, accounting for 18% of all cancer deaths. In 2020, there were approximately 2.2 million new cases and 1.8 million deaths from lung cancer, making it the second most common cancer. The 5-year survival rate for lung cancer patients ranges from 10 to 20%, which is significantly lower compared to other cancers [1]. The most recent cancer statistics report from the United States revealed that lung cancer is the leading cause of cancer deaths for adults aged 50 and over, exceeding the combined deaths caused by breast cancer, prostate cancer, and colorectal cancer [2]. Lung cancer is a complex and heterogeneous disease that is classified into non-small cell lung cancer (NSCLC) and small cell lung cancer based on histological categories. NSCLC is responsible for 85–90% of all lung cancers, with lung adenocarcinoma (LUAD) accounting for approximately 40% of NSCLC cases [3–5]. Despite significant improvements in treatment options, such as surgery, radiation therapy, targeted therapy, and immunotherapy, lung cancer remains an incurable disease for most patients [6, 7] Therefore, understanding the molecular heterogeneity and exploring of the relationship between oncogenes and tumor microenvironment underlying lung cancer will contribute to the development of novel targeted drugs.

Nucleophosmin 3 (NPM3) is a member of the nucleophosmin/nucleoplasmin family and encodes proteins associated with the molecular chaperones nucleoplasmin and nucleophosmin [8]. NPM comprises an N-terminus domain (protein binding), an acidic domain (histone binding) and a C-terminus nucleic acid binding domain. The acidic domain contains multiple potential phosphorylation sites and putative nuclear localization signals. NPM3 was found to be strongly expressed in all 16 human tissues, with particularly strong expression in the pancreas and testis, while the lung exhibited the lowest expression levels. Subcellular fraction analysis showed that NPM3 protein was localized only in the nucleus [9].

During retinoic acid-induced differentiation, NPM3 protein was reduced and the expression level of NPM3 was higher in undifferentiated cells. NPM3 also acts as a chromatin remodeling protein responsible for the unique chromatin structure and replication ability of embryonic stem (ES) cells and facilitates ES cell proliferation [10]. Furthermore, the interaction between NPM3 and transition protein 2 (TP2) is blocked by the histone acetylase p300, altering the DNA condensation properties during mammalian spermiogenesis [11]. Importantly, NPM3 was found to be abundantly expressed in adipose tissue and participated in adipose metabolism. The expression of NPM3 in small extracellular vesicles (sEV-AT) derived from adipose tissue was remarkably reduced in obese individuals compared to lean individuals [12]. NPM3 carried by sEV-AT regulates the PRDM16 mRNA stability, which in turn facilitates white adipose tissue browning [13]. However, the effect of NPM3 on LUAD progression and its relationship with the tumor microenvironment are not yet clear.

In this study, we comprehensively characterized the expression of NPM3 on tumor cells and immune cells in lung adenocarcinoma tissues, and evaluated the correlation between NPM3 expression and clinical prognosis, gene mutations, immune cell infiltration, or immune regulatory genes using multiple biological databases. Then, we attempted to uncover the mechanism by which NPM3 exerts its function using KEGG enrichment analysis. To conclude, we investigated the impact of NPM3 on cell migration or growth using multiple biological functional assays in LUAD cell lines NCI-H1299 and SPC-A1, and profiled the downstream genes regulated by NPM3.

## Materials and methods

### Gene expression, promoter methylation level and protein expression analysis

All database information involved in this study is listed in Table 1. We utilized the UALCAN database to analyze the expression and promoter methylation levels of *NPM3, GLRX3*, and *CALCOCO1* genes in TCGA samples, as well as the protein expression of NPM3 in CPTAC samples [14]. We obtained the immunohistochemical staining data of NPM3 using the HPA database [15]. Furthermore, we analyzed the *NPM3* expression in TCGA and GTEx samples using the GEPIA2 database [16].

**Table 1:** Summary of databases used in this study

Name	Abbreviation	Link
The University of ALabama at Birmingham CANcer data analysis Portal	UALCAN	http://ualcan.path.uab.edu/
Human Protein Atlas	HPA	https://www.proteinatlas.org/
The cBioPortal for Cancer Genomics	cBioPortal	https://www.cbioportal.org/
Sangerbox Database	Sangerbox	http://sangerbox.com/home.html
STRING Database	STRING	https://string-db.org/cgi/input.pl
Gene Expression Profiling Interactive Analysis 2	GEPIA2	http://gepia2.cancer-pku.cn/#index
University of California Santa Cruz	UCSC	https://xenabrowser.net/
Tumor Immune Single-cell Hub 2	TISCH	http://tisch.comp-genomics.org/home/

### Prognosis analysis

We analyzed the relationship between *NPM3* expression and Overall Survival (OS), Disease Free Survival (DFS), Progression Free Interval (PFI) or Disease Free Interval (DFI) using the Sangerbox database [17]. Briefly, we calculated the optimal cut-off values for *NPM3* using the R package maxstat (Maximally selected rank statistics with several *p*-value approximations version: 0.7–25), and patients were divided into high and low *NPM3* expression groups. Prognosis was analyzed using the survfit function of the R package survival [18], and differences in prognosis between the groups were evaluated using the log-rank test method. We also analyzed the influence of *GLRX3*, and *CALCOCO1* on the survival of LUAD patients using the UALCAN database [14].

### Gene expression and mutational landscape

We analyzed the mutation profile of the *NPM3* gene in lung adenocarcinoma using the cBioPortal database [19]. We also analyzed the relationship between *NPM3* expression and the gene mutation landscape using the Sangerbox database [17]. Specifically, we analyzed the mutation landscape in 513 lung adenocarcinoma samples and assessed the variation of mutation frequencies between high and low NPM3 expression group samples using the chi-square test.

### The crosstalk between *NPM3* and immune microenvironment

We investigated *NPM3* expression in immune cells within the LUAD microenvironment at the single-cell level using the TISCH database [20]. Furthermore, we analyzed the relationship between *NPM3* expression and immune cell infiltration or immune-related genes using the Sangerbox database [17]. To do this, we used the ESTIMATE algorithm to evaluate the relationship between *NPM3* expression and ESTIMATE score, immune score, or stromal score [21], the relationship between *NPM3* expression and immune cell infiltration was analyzed using TIMER [22], EPIC [23] or QUANTISEQ [24] algorithms. Finally, we used Spearman's correlation to assess the relationship between *NPM3* and immune modulator (immune checkpoint, chemokine, chemokine receptor, MHC) expression. Briefly, we downloaded the uniformly normalized dataset from the UCSC (https://xenabrowser.net/) database, and extracted the expression data of *ENSG00000107833 (NPM3)* gene in each LUAD sample. We then performed log2(x + 0.001) transformation on the gene expression data and mapped the gene expression profile to Gene Symbol. Finally, we reassessed the immune cell infiltration score of each patient based on gene expression using the TIMER [22], EPIC [23] or QUANTISEQ [24] method of R package IOBR [25].

### Co-expressed genes and KEGG enrichment analysis

We obtained 608 positively and 189 negatively associated genes (absolute value of Pearson r ≥ 0.3) with *NPM3* using the UALCAN database [14], and performed KEGG enrichment analysis [26] for each of these two gene sets. Briefly, we used the KEGG rest API (https://www.kegg.jp/kegg/rest/keggapi.html) to obtain the latest KEGG Pathway gene annotations as background, mapped the genes to the background set, and the R package clusterProfiler [27] was used for enrichment analysis to obtain the results of gene set enrichment. A minimum gene set was set at 5 and a maximum gene set at 5000, *p* value < 0.05 and FDR < 0.25 were considered statistically significant.

### Cell culture and small interfering RNA (siRNA) transfection

We purchased NCI-H1299, SPC-A1 lung adenocarcinoma cell lines from the Chinese Academy of Sciences Shanghai Cell Bank. They were cultured in a constant temperature incubator containing 5% CO_2_ at 37 ℃ as required. Small interfering RNA transfection procedures were performed according to the instructions of the GP-transfect-Mate transfection reagent (GenePharma, Shanghai, China). The siRNA used in this study was purchased from KeyGEN BioTECH (Nanjing, China) and the sequences are siNPM3#1 sense, AGGUAGAGGAAGAGGAUGATT, antisense, UCAUCCUCUUCCUCUACCUTT; siNPM3#2 sense, GGACAGUGAUGAGGAAGAATT, antisense, UUCUUCCUCAUCACUGUCCTT.

### RNA extraction, reverse transcription and real-time fluorescence quantitative PCR (qPCR) experiments

The RNA extraction procedure in this study was performed according to the manufacturer's instructions of the TransZol Up Plus RNA Kit (TransGen Biotech #ER501, Beijing, China). Briefly, lung adenocarcinoma cells were collected using TRizol up, RNA was isolated and cleaned using several reagents supplied as part of the kit, and RNA concentration and purity were measured. The reverse transcription experiments were performed referring to the manufacturer's instructions of HiScript III All-in-one RT SuperMix Perfect for qPCR (Vazyme, #R333, Nanjing, China). The qPCR experiments were performed on the Applied Biosystems® 7500 Fast (Applied Biosystems, USA) using Taq Pro Universal SYBR qPCR Master Mix (Vazyme #Q712, Nanjing, China). The primer sequences used in this study are listed in Supplementary Table S1.

### Cell cloning experiments and CCK-8 experiments

After transfection of siRNA for 48 h in NCI-H1299 and SPC-A1 cells, 1000 cells were collected for clone formation assay, and clone size was observed after 14 days. Cells were fixed with paraformaldehyde and stained with 0.1% crystal violet.

After transfection of siRNA for 48 h in NCI-H1299 and SPC-A1 cells, 2000 cells were collected for CCK-8 experiments, three replicate wells were set up, the absorbance at 450 nm was measured using CCK-8 solution on 0, 2, 4, 6 days respectively, and the cell growth rate was calculated.

### Cell scratching experiments and transwell experiments

Cell scratching experiments were performed following transfection of siRNA for 48 h in NCI-H1299 and SPC-A1 cells, and cell migration was recorded by microscopy at 0 h, 18 h, and 24 h, respectively.

For transwell experiments, siRNA was transfected for 48 h in NCI-H1299 and SPC-A1 cells. Subsequently, 100 µl of medium containing 2*10^4 cells with 10% serum was added to the upper chamber of the transwell, and 600 µl of medium with 20% serum was added to the lower chamber of the transwell. After 24 h, cells were fixed using anhydrous ethanol, stained using 0.1% crystal violet, and photographed under the microscope to record the staining.

### Statistical analysis

Analyses and graphical presentation were performed using the GraphPad Prism 8.0 software. Unless stated otherwise, single comparison was performed by a two tailed Student’s t test, multiple comparisons were analyzed by One-way ANOVA. The Mann–Whitney U test was used for non-normal distribution. The patient survival curves were presented by the Kaplan–Meier method and the difference was determined using a log-rank test. Differences in the frequency of gene mutations among high and low *NPM3* expression samples were assessed using a chi-square test. Pearson's correlation coefficient was used to determine the relationship between gene expression and immune infiltration. Spearman's correlation coefficient was used to assess gene expression correlation. Differences with * *P* < 0.05, ** *P* < 0.01, and *** *P* < 0.001 were considered significant.

## Results

### The NPM3 expression and its relationship with clinical parameters in LUAD

**Fig. 1:**
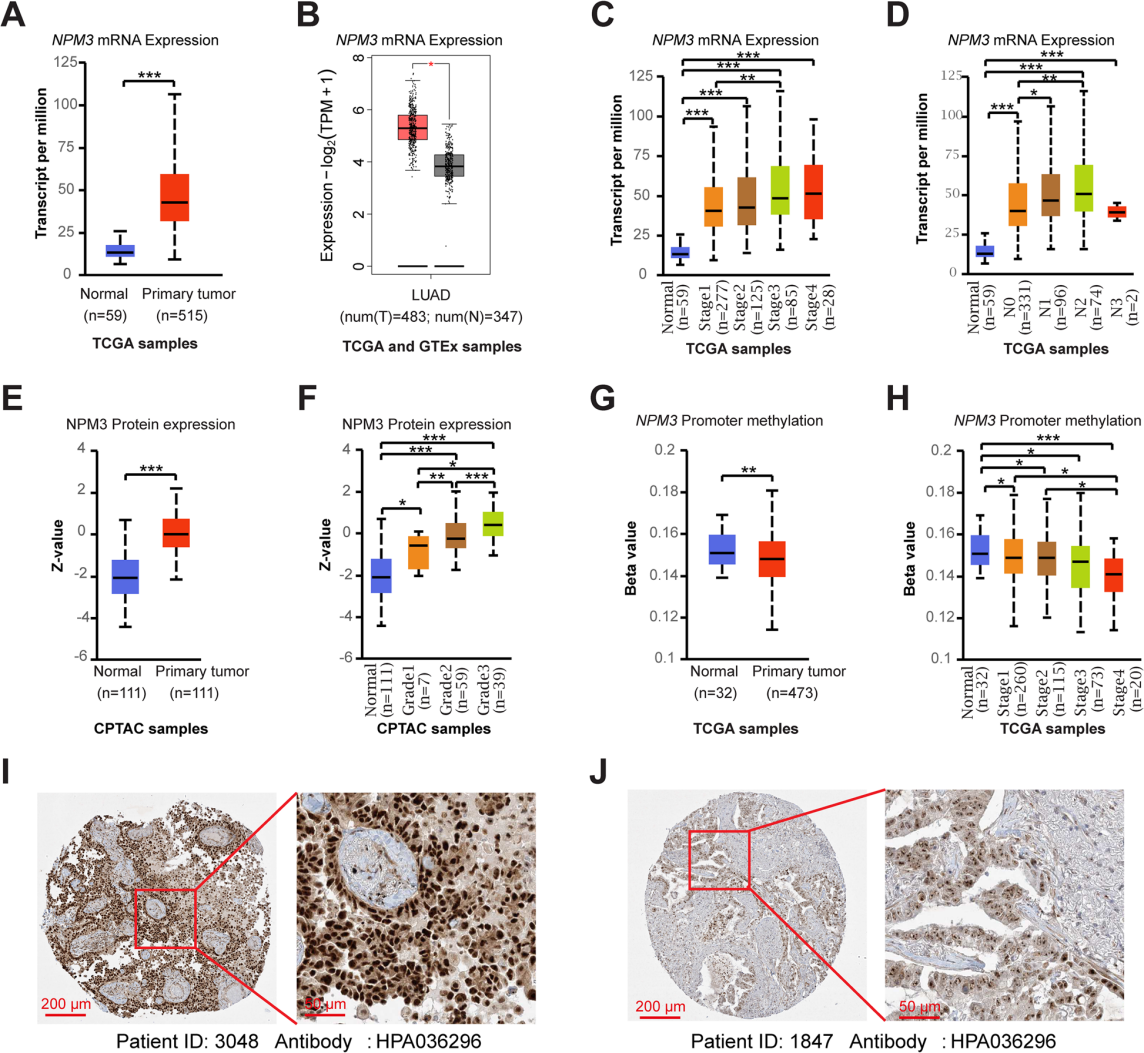
NPM3 expression characteristics in LUAD. **A**-**B** *NPM3* mRNA expression in TCGA-LUAD and GTEx-lung samples. **C**-**D** *NPM3* mRNA expression in LUAD with different pathological parameters. **E** NPM3 protein expression is elevated in CPTAC-LUAD samples. **F** NPM3 protein expression in grade. **G** NPM3 promoter methylation levels are reduced in TCGA-LUAD samples. **H** *NPM3* promoter methylation levels are reduced in higher stage. **I**-**J** Immunohistochemical staining of NPM3 protein in LUAD tissue slides. The Mann–Whitney U test was used to assess the significance of observed differences. * P < 0.05, ** *P* < 0.01, and *** *P* < 0.001 were considered significant

To determine the differences of NPM3 expression between normal and tumor tissues, we analyzed the *NPM3* mRNA expression in TCGA-LUAD and GTEx samples using the UALCAN and GEPIA2 databases. We found that *NPM3* mRNA expression was significantly elevated in LUAD tissues (Fig. 1A-B). We additionally investigated the association between *NPM3* and clinical parameters, and the results indicated that *NPM3* mRNA expression was significantly elevated in patients with advanced clinical Stage and N staging (Fig. 1C-D), irrespective of patients' gender, age, race and smoking habits (Supplementary Figure S1). We further analyzed the promoter methylation level of *NPM3* in TCGA-LUAD samples using the UALCAN database. The results indicated that the promoter methylation level of *NPM3* was significantly decreased in tumors and negatively correlated with the clinical Stage of patients (Fig. 1E-F), implying that the elevated mRNA expression of *NPM3* appeared potentially driven by the decreased promoter methylation level. More importantly, we analyzed NPM3 protein expression utilizing the UALCAN and HPA databases. We found that NPM3 protein expression was significantly elevated and positively correlated with clinical Grade in CPTAC-LUAD samples (Fig. 1G-H). Immunohistochemical staining results revealed that NPM3 protein is expressed in the nucleus (Fig. 1I-J). In summary, NPM3 is highly expressed and positively correlated with tumor malignancy in LUAD.

### *NPM3* is associated with worse clinical prognosis

**Fig. 2:**
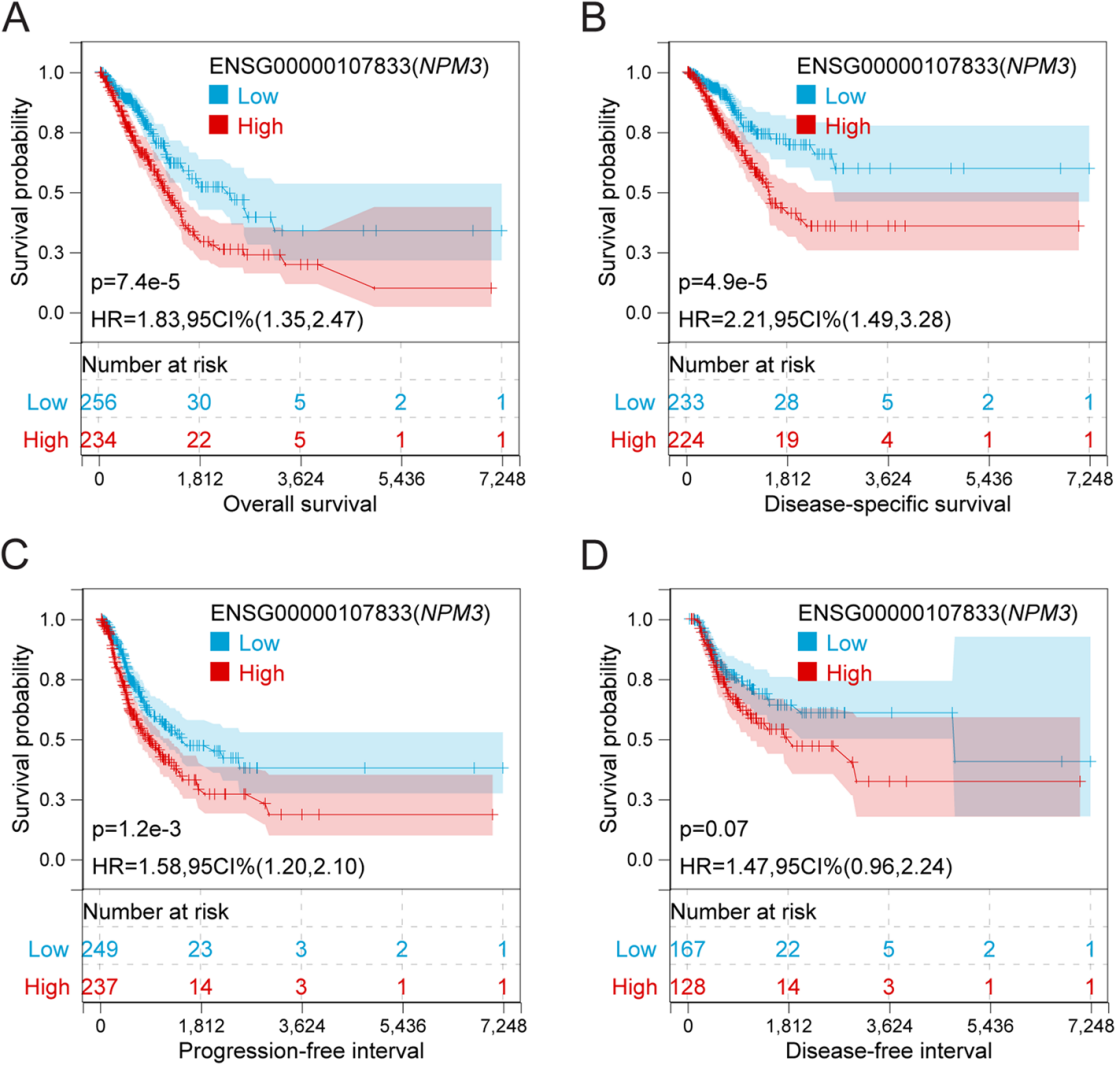
Effect of *NPM3* expression on prognosis. **A**-**D** The association of *NPM3* expression with OS, DSS, PFI, and DFI. *P* values were determined by log-rank test

Based on the significantly elevated expression of NPM3 in LUAD samples and its positive correlation with tumor malignancy, it is possible that NPM3 may have a negative impact on patient survival. To investigate this possibility, we categorized patients into high and low *NPM3* expression groups and analyzed their prognosis using the Sangerbox database. The logrank test was used to compare the differences in prognosis between the two groups. Our results indicated that high *NPM3* expression was significantly associated with poor prognosis in LUAD, as demonstrated by Kaplan–Meier curves for overall survival (OS, HR = 1.83, *p* = 7.4e-5) (Fig. 2A), disease-free survival (DFS, HR = 2.21, *p* = 4.9e-5) (Fig. 2B), progression-free interval (PFI, HR = 1.58, *p* = 1.2e -3) (Fig. 2C), and disease-free interval (DFI, HR = 1.47, *p* = 0.07) (Fig. 2D). These findings suggest that *NPM3* has potential as a prognostic biomarker in LUAD.

### *NPM3* expression and mutation landscape

**Fig. 3:**
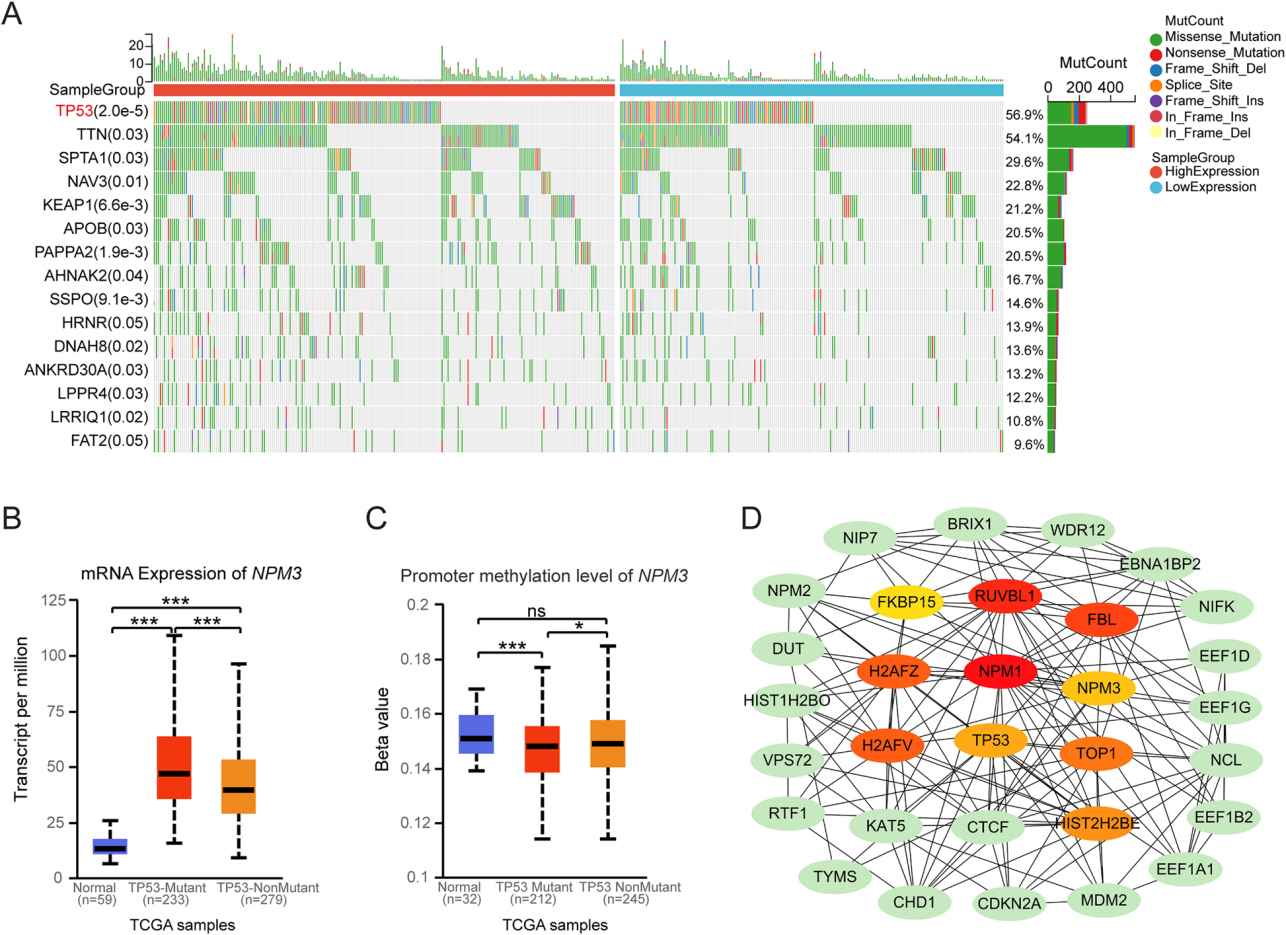
Relationship between NPM3 expression and gene mutation landscape. **A** Relationship between *NPM3* expression and gene mutation landscape was analyzed through the Sangerbox database. **B**-**C** *NPM3* mRNA expression and promoter methylation levels upon TP53 mutation were analy zed through the UALCAN database. **D** The interacting protein network of NPM3 was analyzed through the STRING database and Cytoscape 3.9.1 software. The top ten important nodes are highlighted based on Betweenness Centrality parameter. Chi-square test was used to assess differences in the frequency of gene mutations. The Mann–Whitney U test was used to assess the significance of observed differences. * P < 0.05 and *** *P* < 0.001 were considered significant, ns, no significance

We first analyzed the somatic mutation of *NPM3* using the cBioPortal database. The results revealed that *NPM3* exhibits a low somatic mutation rate in LUAD, indicating that the abnormal expression of *NPM3* is mainly due to epigenetic modification rather than genetic alteration (Supplementary Figure S2). We further investigated the relationship between *NPM3* expression and mutation landscape, and found that the mutation frequencies of *TP53* (*p* = 2.0e-5), *TTN* (*p* = 0.03), *SPTA1* (*p* = 0.03), *NAV3* (*p* = 0.03), or *KEAP1* (*p* = 0.03) genes were higher in high *NPM3* expression samples (Fig. 3A). Notably, TP53 is a well-known tumor suppressor, and *TP53* mutations are capable of inducing carcinogenesis, tumor development, resistance to therapy, and influencing patient prognosis and responsiveness to therapy [28]. To confirm the association between NPM3 expression and TP53 mutation, we analyzed *NPM3* expression in the presence of *TP53* mutation using the UALCAN database. The results showed that *NPM3* mRNA expression was higher (Fig. 3B) and promoter methylation level was lower (Fig. 3C) in the presence of TP53 mutation, consistent with the results of Fig. 3A. Furthermore, we retrieved the interaction protein network of NPM3 protein using STRING database [29], and obtained a network comprising 31 nodes with 156 edges, we downloaded and imported this network into Cytoscape 3.9.1 software, and the central hub genes were identified depending on the Betweenness Centrality (BC) value, degree, and Closeness Centrality (CC) value using CytoHubba (Supplementary Table S2). The nodes with high BC in the network often serve as an important bridge for information transfer in the network. We identified the top 10 important nodes in the network based on the Betweenness Centrality parameter, including NPM3, NPM1, TP53, RUVBL1, FBL, H2AFV, H2AFZ, TOP1, HIST2H2BE, FKBP15 (Fig. 3D and Supplementary Table S2), suggesting a potential functional interaction between NPM3 and TP53.

### Single-cell level profiling of *NPM3* expression signatures on LUAD microenvironment cells

**Fig. 4:**
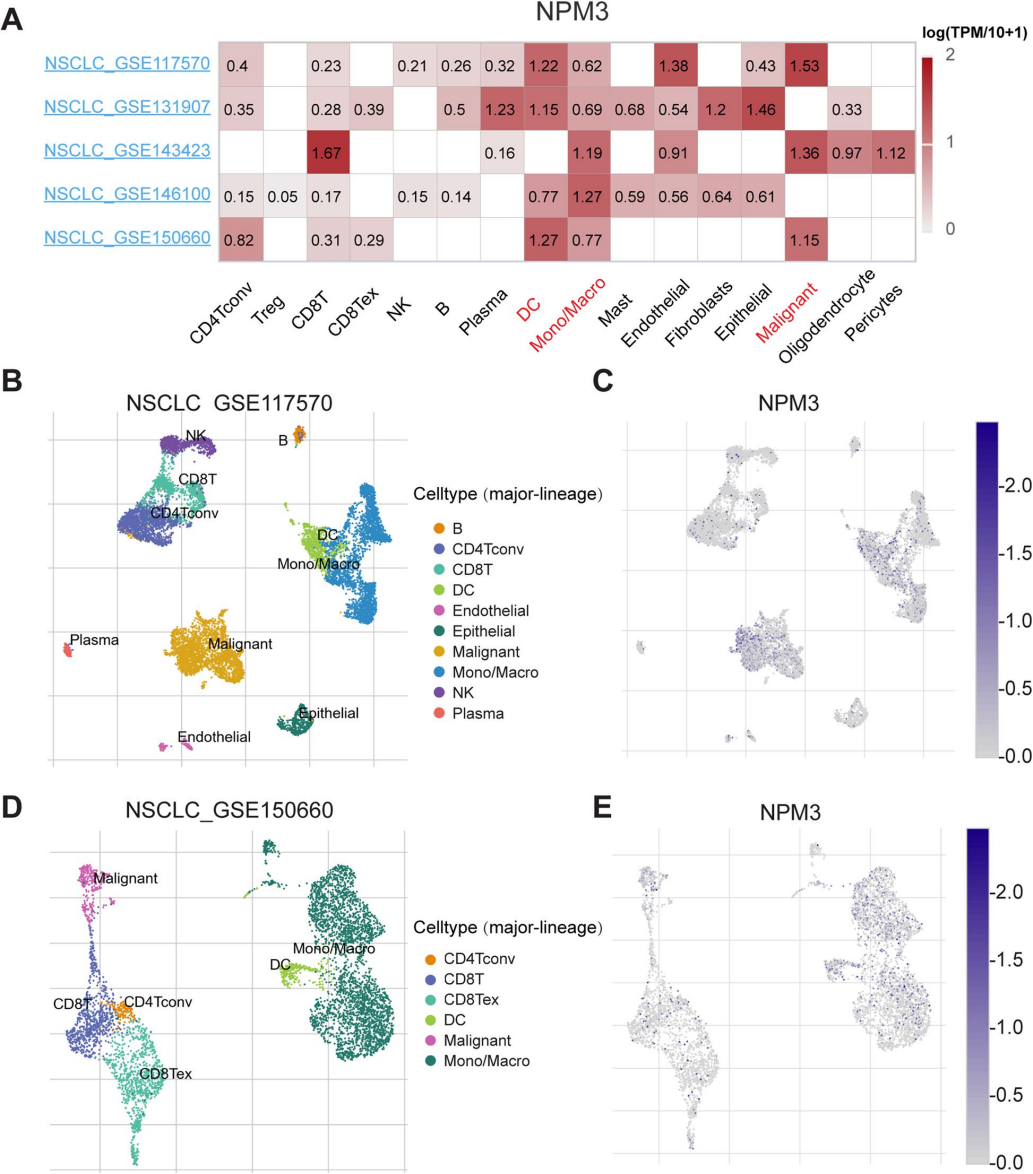
*NPM3* expression at the single cell level. **A** Heatmap displaying the *NPM3* expression in different cell types from different NSCLC databases. **B**-**C** Single-cell clustering plot and *NPM3* expression in the GSE117570 dataset. **D**-**E** Single-cell clustering plot and *NPM3* expression in the GSE150660 dataset. CD4Tconv means conventional CD4 T cells. CD8Tex means exhausted CD8 T cells

We investigated the *NPM3* expression in LUAD microenvironment cells at the single cell level using the TISCH database (Fig. 4A). The results demonstrated that *NPM3* was predominantly expressed in dendritic cells (DC) and monocytes/macrophages (Mono/Macro) clusters in GSE117570 (Fig. 4B-C), GSE131907, GSE143423, GSE146100 (Supplementary Figure S3A-C), and GSE150660 (Fig. 4D-E). Moreover, in GSE143423, *NPM3* was also expressed mainly in the CD8T cell cluster (Fig. 4A). In GSE131907, *NPM3* was mainly expressed in Plasma, Fibroblasts and Epithelial cell clusters (Fig. 4A). These findings suggest that NPM3 might also play a functional role in immune cells or stromal cells, in addition to its role in cancer cells.

### Relationship between tumor-infiltrating immune cells and *NPM3* expression

**Fig. 5:**
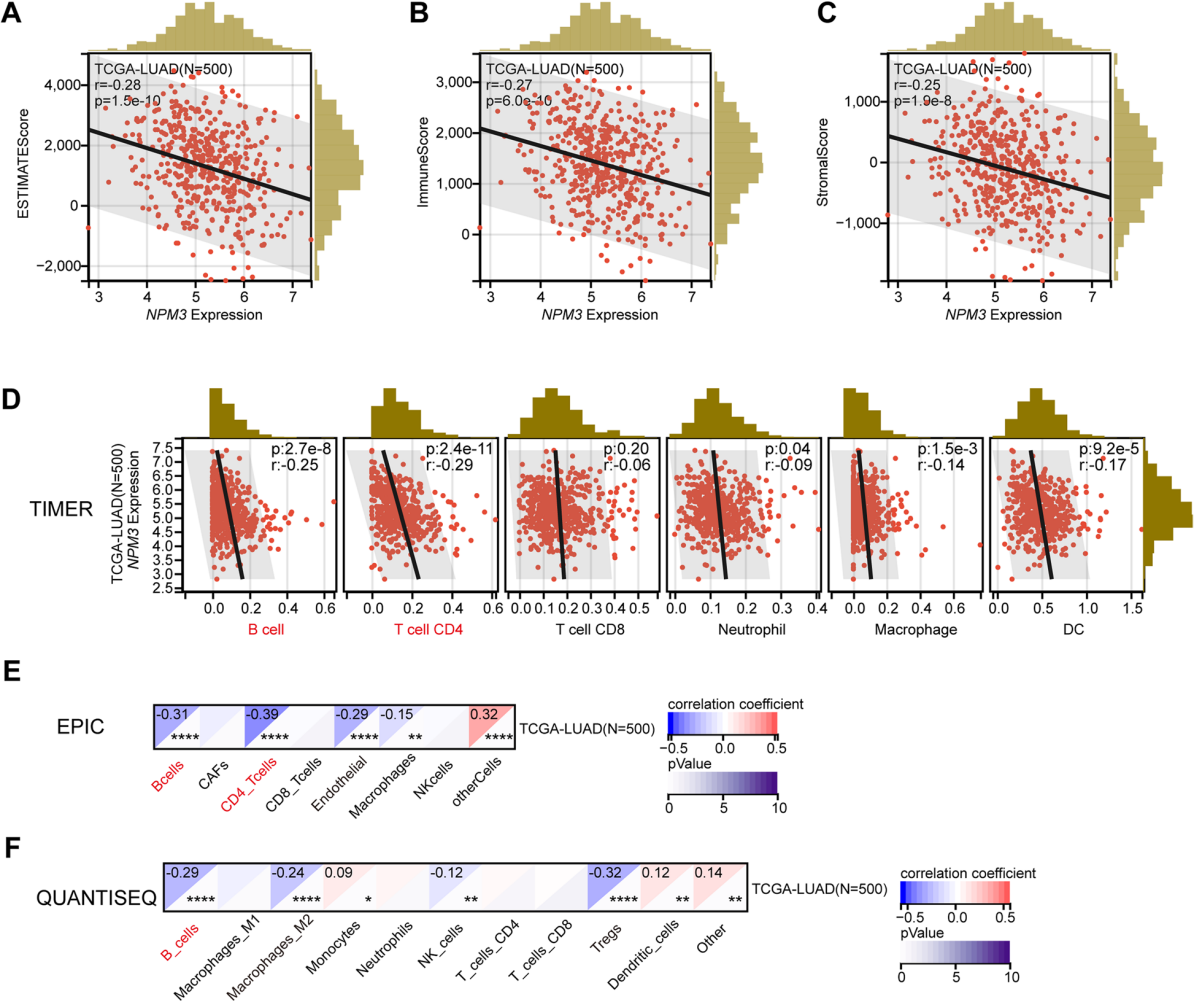
Codependence of *NPM3* expression and immune microenvironment. **A**-**C** The relationship between *NPM3* expression and ESTIMATE score, immune score or stromal score. **D**-**F** the relationship between *NPM3* expression and immune cell infiltration was analyzed using TIMER, EPIC or QUANTISEQ algorithms. Pearson's correlation coefficient was used to determine the relationship between gene expression and immunescore or immune cell infiltration

Previous studies have demonstrated that the type, density and dysfunction of tumor-infiltrating lymphocytes influences the survival of cancer patients [30, 31]. We first evaluated the relationship between *NPM3* expression and immune score using the ESTIMATE algorithm. The results revealed a significant negative correlation between *NPM3* expression and ESTIMATE score (Fig. 5A), immune score (Fig. 5B), and stromal score (Fig. 5C), suggesting that tumors with high *NPM3* expression likely suffer from immunosuppression.

Next, we proceeded to determine whether *NPM3* expression was associated with immune cell infiltration in LUAD using various algorithms. As a result, *NPM3* expression was negatively correlated with B cells (cor = -0.25, cor = -0.31, cor = -0.29), as illustrated by the TIMER, EPIC and QUANTISEQ algorithms (Fig. 5D-F). Furthermore, *NPM3* expression was significantly negatively correlated with CD4 + T cells (cor = -0.29, cor = -0.39) (Fig. 5D-E) and endothelial cells (cor = -0.29) (Fig. 5E). *NPM3* expression was also significantly negatively correlated with M2 macrophages (cor = -0.24) and Tregs cells (cor = -0.32) (Fig. 5F). However, there was no significant correlation between CD8 + T cells and *NPM3* expression (*p* > 0.05). These findings suggest that NPM3 plays an essential role in regulating immune infiltration in LUAD.

### The association between *NPM3* expression and immune-related genes

**Fig. 6:**
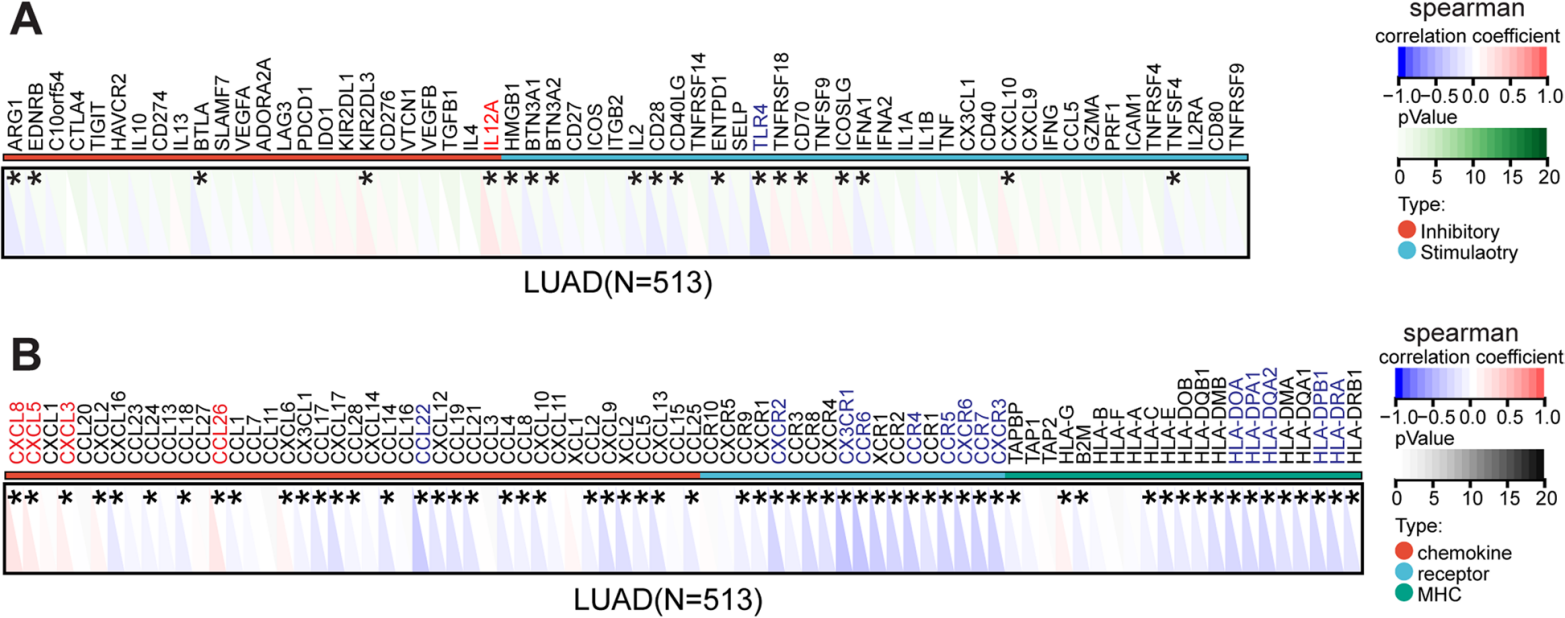
Correlation between *NPM3* expression and immune-related genes in LUAD. **A** Heat map illustrates the correlation between *NPM3* and immune checkpoints. **B** Heat map illustrates the correlation between *NPM3* and chemokines, chemokine receptors, MHC. Gene expression correlation was analyzed using Spearman's rank correlation coefficient

Immune checkpoints are vital in the tumor immune microenvironment, and directly mediate the anti-tumor immune response of the host [32]. Within this context, we performed a correlation analysis between *NPM3* and immune checkpoints (Fig. 6A). The results demonstrated that *IL12A,* an immunosuppressive checkpoint, showed the strongest positive correlation with *NPM3* expression, while *TLR4*, an immunostimulatory checkpoint, showed the strongest negative correlation with *NPM3* expression. Furthermore, we investigated the correlation between *NPM3* expression and immune-related genes (including chemokines, chemokine receptors, and MHC genes. Our analysis revealed that *NPM3* expression was positively correlated with chemokines and chemokine receptors such as *CXCL8, CXCL5,* and *CCL26*, while *NPM3* expression was negatively correlated with several immunomodulatory genes, including *CCL22, CXCR2, CX3CR1, CCR6, HLA-DOA*, and *HLA-DQA2* (Fig. 6B).

### The potential mechanism of NPM3 is explored in LUAD

**Fig. 7:**
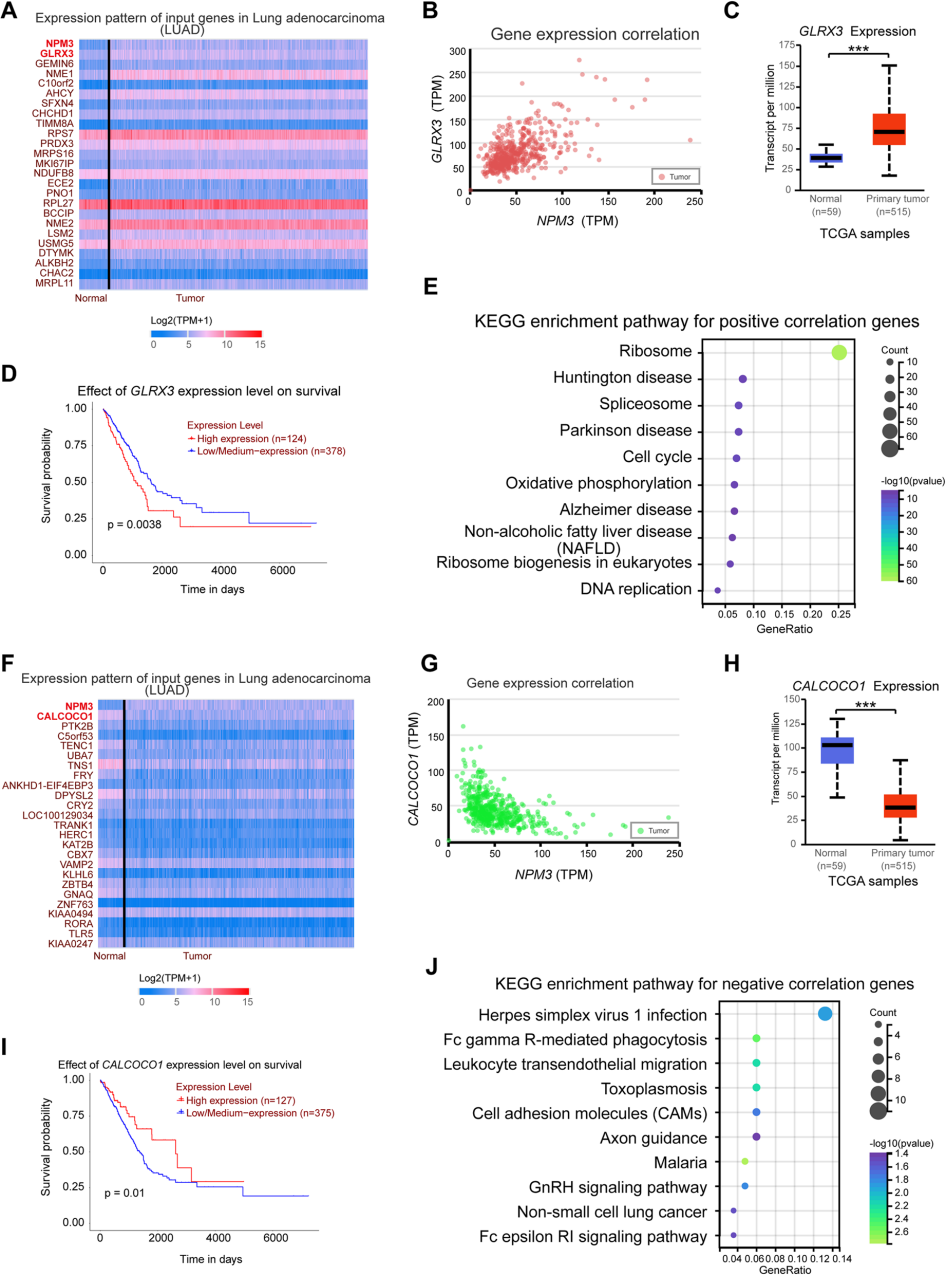
The potential mechanism of NPM3 in LUAD. **A** Heat map plotting of genes positively correlated with *NPM3* expression. **B** Correlation between *NPM3* and *GRLX3* expression. **C** *GRLX3* mRNA expression in TCGA-LUAD samples. **D** Effect of *GRLX3* expression on LUAD prognosis. **E** The KEGG enrichment pathway for positive correlated genes. **F** Heat map plotting of genes negatively associated with *NPM3* expression. **g** Correlation between *NPM3* and *CALCOCO1* expression. **H** *CALCOCO1* mRNA expression in TCGA-LUAD samples. **I** Effect of *CALCOCO1* expression on LUAD prognosis. **J** The KEGG enrichment pathway for negative associated genes. The Mann–Whitney U test was used to assess the significance of observed differences (**C** and **H**). *** *P* < 0.001 were considered significant. *P* values were determined by log-rank test (**D** and **I**)

To investigate the potential mechanism of NPM3 in LUAD, we obtained 608 genes positively associated with *NPM3* (Pearson r ≥ 0.3) from the UALCAN database (Supplementary Table S3), with *GLRX3* being the most correlated gene (Fig. 7A-B). Further analysis showed that *GLRX3* was significantly overexpressed in LUAD (Fig. 7C) and high *GLRX3* expression was associated with poor prognosis (Fig. 7D), consistent with previous reports that *GLRX3* is preferentially expressed in lung cancer [33]. KEGG enrichment analysis of these 608 genes showed significant enrichment in pathways related to ribosome, spliceosome, cell cycle, Parkinson's disease, DNA replication, Huntington's disease, and nonalcoholic fatty liver disease (NAFLD) (Fig. 7E, Supplementary Table S4). We also obtained 189 genes negatively associated with *NPM3* (Pearson r ≤ -0.3) from the UALCAN database (Supplementary Table S5), with *CALCOCO1* being the most negatively associated gene (Fig. 7F-G). We found a significant decrease in *CALCOCO1* expression in LUAD (Fig. 7H) and an association between low *CALCOCO1* expression and poor prognosis (Fig. 7I), consistent with previous reports that CALCOCO1 acts synergistically with CCAR1 to co-activate the tumor suppressor TP53 [34]. KEGG enrichment analysis of these 189 genes revealed significant enrichment in pathways related to malaria, Fc gamma R-mediated phagocytosis, leukocyte transendothelial migration, toxoplasmosis, GnRH signaling pathway, cell adhesion molecules (CAMs), non-small cell lung cancer, and Fc epsilon RI signaling pathway pathways (Fig. 7J, Supplementary Table S6).

### NPM3 knockdown inhibits cell proliferation in LUAD cells NCI-H1299 and SPC-A1 in vitro

**Fig. 8:**
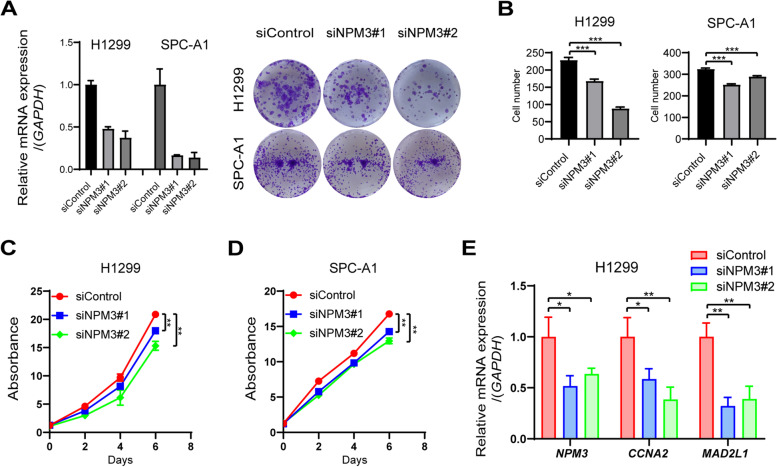
NPM3 knockdown inhibits proliferation of LUAD cells. **A** Cell clone formation assay confirms the effect of NPM3 knockdown on LUAD cell growth. **B** Statistical analysis of A plots. **C**-**D** CCK-8 assay confirms the effect of NPM3 knockdown on proliferative capacity of LUAD cells. **E** The mRNA expression of CCNA2 and MAD2L1 were analyzed by qPCR assay after NPM3 knockdown. Data represent the mean ± SD. * P < 0.05, ** *P* < 0.01, and *** *P* < 0.001. *P* values were determined by one-way ANOVA

To investigate the role of NPM3 in lung adenocarcinoma, we examined the effect of NPM3 on cell proliferation. First, we performed cell clone-formation assays and found that NPM3 knockdown significantly inhibited the cell clone-forming ability in NCI-H1299 cells compared to controls (Fig. 8A-B). The consistent results were observed in SPC-A1 cells (Fig. 8A-B). In addition, we performed CCK-8 experiments, which showed that NPM3 knockdown significantly inhibited the cell proliferation capacity in either NCI-H1299 or SPC-A1 cells compared to controls (Fig. 8C-D). These results indicated that suppression of NPM3 could retard LUAD cell proliferation in vitro. Since *NPM3* was positively associated with certain genes in the cell cycle pathway (including *MAD2L1, CCNA2, BUB3, CCNB2, DBF4, CDK1* and *CCNB1*) (Fig. 7E), we attempted to explore the regulatory relationship between them. As a result, we found that *NPM3* knockdown significantly suppressed the mRNA expression of *CCNA2* and *MAD2L1* (Fig. 8E). The above results suggest that NPM3 knockdown may inhibit LUAD cell growth by suppressing CCNA2 and MAD2L1 expression.

### NPM3 knockdown inhibits cell migration in LUAD cells NCI-H1299 and SPC-A1 in vitro

**Fig. 9:**
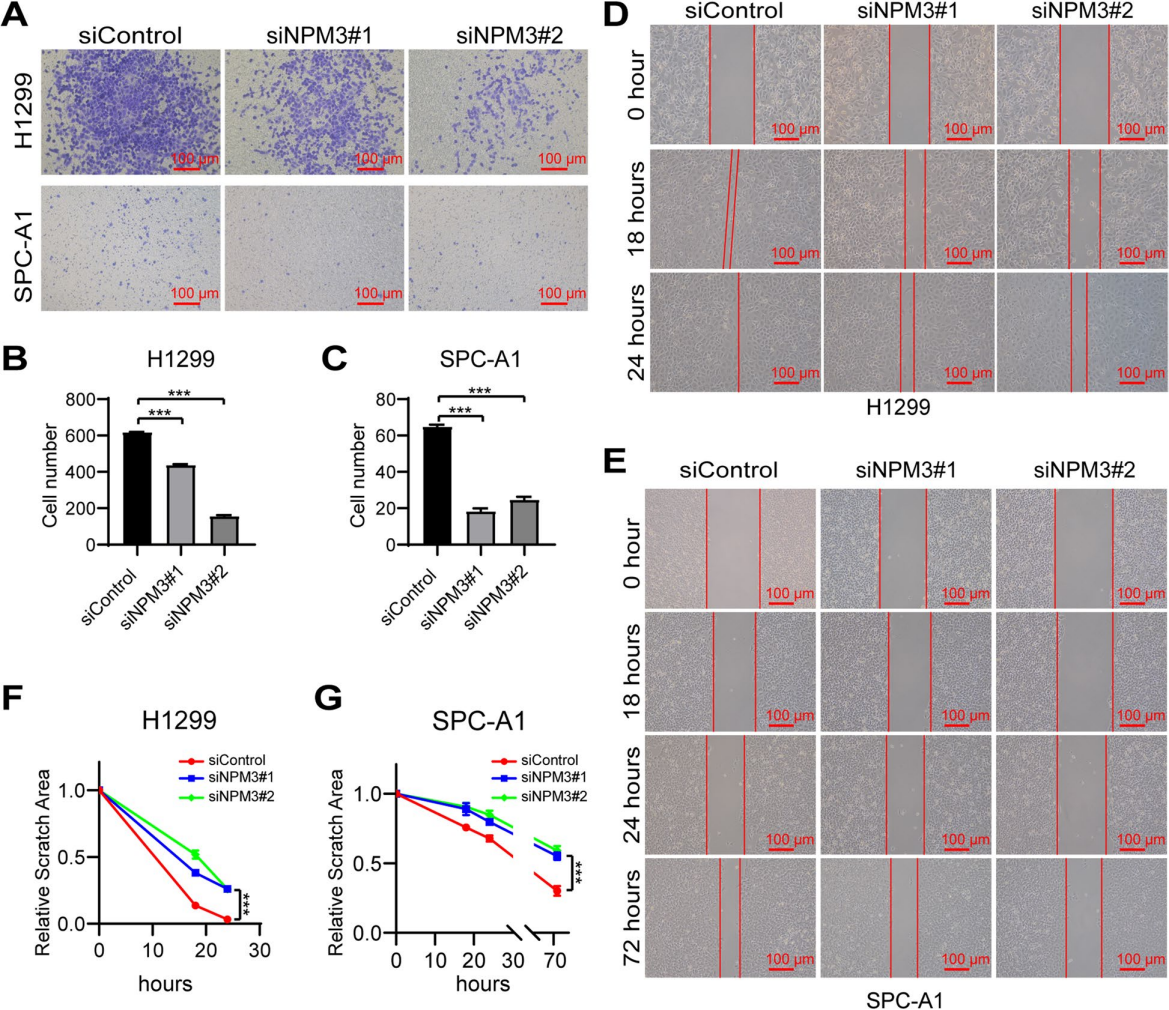
NPM3 knockdown inhibits migration of LUAD cells. **A** The effect of NPM3 knockdown on the migratory ability of LUAD cells was analyzed by transwell assay. **B**-**C** The count of migrating cells. **D**-**E**. The effect of NPM3 knockdown on the migratory ability of LUAD cells was analyzed by cell scratch assay. F-G. The statistics of scratch healing area. Data represent the mean ± SD. * P < 0.05, ** *P* < 0.01, and *** *P* < 0.001. *P* values were determined by one-way ANOVA

To investigate the effect of NPM3 on cell migration ability, we performed cell scratch assay and transwell assay. Firstly, the results of transwell assays showed that NPM3 knockdown significantly inhibited the cell migration ability in NCI-H1299 or SPC-A1 cells compared to the control (Fig. 9A-C). In addition, the results of cell scratch assays revealed that the scratch closure rate was significantly slower in the NPM3 knockdown group than in the control group (Fig. 9D-G). These findings suggest that suppression of NPM3 can significantly inhibit the migration of LUAD cells in vitro.

## Discussion

Lung adenocarcinoma (LUAD) is one of the most common malignancies. Sourcing critical targets for LUAD diagnosis and treatment is emerging as a sought-after investigation area following the rapid development of molecular biology. As such, gaining adequate insight into the molecular pathways that manipulate LUAD progression and prognosis has become an essential objective. This study reveals that NPM3 is a prognostic risk factor in patients and that its mRNA and protein expression are elevated in LUAD. Additionally, in the immune microenvironment, NPM3 is expressed mainly on DC and Mono/Macro immune cells. NPM3 expression is negatively correlated with immune scores, immune infiltration of B cells and CD4 T cells. Finally, NPM3 inhibition suppressed the proliferation and migration of LUAD cells, presumably by regulating the cell cycle and CAMs pathways. In conclusion, our investigations are based on numerous biological database findings, and the results have been validated within in-vitro experiments.

The Nucleophosmin/Nucleoplasmin (NPM) family proteins are termed molecular (or nuclear) chaperones mediating the ordered assembly of proteins, allowing subdivision into four groups based on protein sequence: NPM1, NPM2, NPM3, and invertebrate NPM proteins [35]. We barely know how NPM3 acts in tumors. There is only one report implicating that NPM3 might engage in cancer proliferation as a novel target of transcription factor SP1 in Hela cells [36]. However, NPM1 has attracted substantial attention lately due to its multiple functions, including nucleoplasm-nucleolus shuttling, chromatin remodeling, DNA replication, and mRNA transcription. In particular, NPM1 serves as a double-edged sword in tumors. NPM1 protein expression is upregulated in colorectal and hepatocellular carcinoma, promoting tumor migration and invasion. In contrast, NPM1 protein expression is decreased in gastric cancer, inhibiting tumor proliferation and migration, and thus acting as a tumor suppressor [37–39]. More interestingly, several reports hinted at a synergistic action of NPM3 with NPM1, NPM3 enhances the nucleoplasm-nucleolus shuttling activity of NPM1 through inhibition of NPM1's RNA binding activity in somatic cells [40]. NPM3 tremendously enhances transcription in the cellular system by suppressing the histone assembly activity of NPM1 in vitro [41]. In addition, NPM3 interacts with NPM1 to inhibit ribosome biogenesis [42]. Our study demonstrates for the first time that NPM3 mRNA and protein expression is elevated in LUAD with reduced promoter methylation levels using multiple databases. NPM3 is a risk factor for clinical prognosis in LUAD patients. We also identified a strong association between *NPM3* expression and *TP53* mutations. Finally, we demonstrated that NPM3 knockdown inhibits the proliferation and migration of LUAD cells in a cellular model, indicating that NPM3 is a potential novel target for LUAD.

The development of lung cancer is a complex process that involves interactions among tumor cells, stromal fibroblasts, and immune cells. Despite the advances in cancer immunotherapy, effective predictors and favorable response rates remain unavailable [43–46]. Single-cell RNA sequencing (scRNA-seq) has helped to improve our understanding of the various cell states in the tumor microenvironment. In this study, single-cell analysis revealed that *NPM3* is expressed not only in malignant cells but also in immune cells (DC and Mono/Macro cells) and stromal cells (e.g. fibroblasts) (Fig. 4). This is the first report of *NPM3* expression in various cell clusters used for cancer research. DC and Mono/Macro are involved in the intrinsic immune response, additionally, their role as antigen-presenting cells presenting tumor antigens to T cells is essential for an effective T cell-mediated antitumor response [47]. These findings suggest that NPM3 may exert its influence on tumor progression by affecting intrinsic immunity and adaptive immunity through DC and Mono/Macro.

Furthermore, we evaluated the cross-talk between *NPM3* expression and the immune microenvironment using multiple algorithms. We found that *NPM3* expression was mainly negatively correlated with the infiltration of B cells and CD4 T cells, and somewhat negatively correlated with the infiltration of DCs and macrophages, but the correlation coefficient was low. While regarding a negative correlation between NPM3 expression and M2 tumor-associated macrophages that was observed in Fig. 5F, we think that this exactly indicates a very complicated role of NPM3 in the tumor microenvironment. We believe that NPM3 plays a more important role in the initial stage of tumor immunity and immune regulation, rather than in the effector phase. CD4 T cells are the cornerstone of antitumor immunity, mediating antitumor immunity primarily by contributing to CD8 cytotoxic T lymphocytes (CTL) and antibody responses, and by secreting effector cytokines such as interferon-γ (IFNγ) and tumor necrosis factor-α (TNFα) [48]. B cells can capture tumor antigens and activate immune cells in the initial stage of the immune response, or participate in tumor killing by secreting antibodies, such as CCL2, CXCR4, CCL5, CXCL5 and CXCL10, which trigger the activation of CD4 and CD8 T cells [49]. The results in Fig. 6B show that *NPM3* expression was negatively correlated with *CCL2, CXCR4, CCL5* or *CXCL10*, and positively correlated with *CXCL5*. In addition, tumor infiltrating B cells exerts anti-tumor immunity by secreting tumor-specific antibodies, facilitating T-cell responses, and maintenance the structure and function of tertiary lymphoid structures, which are all responsible for the beneficial outcome of lung cancer [50–53]. However, B cells acting as a multifaceted effector are able to evolve into an immunosuppressive phenotype secreting IL-35, also called regulatory B cells, resulting in tumor progression [51, 53]. Collectively, a comprehensive analysis of immune cell infiltration, immune checkpoint expression, and cell function experimental results suggests that high NPM3 expression is more probably mediating the immunosuppressive microenvironment to promote tumor progression.

To further elucidate the mechanism of NPM3 action in LUAD cells beyond its effects on the immune microenvironment, we investigated the potential signaling pathways in which *NPM3*-associated genes are involved. Our results revealed a positive association between *NPM3* expression and genes involved in cell cycle signaling pathways, such as *MAD2L1, CCNA2, BUB3, CCNB2, DBF4, CDK1*, and *CCNB1* (Supplementary Table S4). Previous studies have shown that FOXM1 reduces the expression of CCNA2 and CCNB1, thereby promoting the proliferation of LUAD cells [54]. TRAP1 modulates the expression of MAD2L1, CDK1, and CCNB1 thereby promoting cell cycle progression and mitotic entry [55]. Additionally, miR-139-5p inhibits LUAD cell proliferation, migration, and invasion by targeting MAD2L1 [56]. Our results demonstrated that NPM3 knockdown suppressed the expression of CCNA2 and MAD2L1, revealing the molecular mechanism by which NPM3 promotes LUAD cell proliferation. Furthermore, we found a negative correlation between *NPM3* and the NSCLC pathway *RASSF5* gene (Supplementary Table S6), which acts as a tumor suppressor by stabilizing Rb and nuclear P53 [57]. Overall, we found that NPM3 was positively associated with genes that promote tumor proliferation, with NPM3 positively regulating the expression of *CCNA2* and *MAD2L1*, and *NPM3* was negatively correlated with the tumor suppressor *RASSF5*.

In this study, we comprehensively investigated the expression and function of NPM3 in LUAD and its relationship with the tumor microenvironment. However, some limitations remain. Firstly, more clinicopathological tissues are required to validate NPM3 expression. Secondly, the function of NPM3 was only investigated in LUAD cells and its role in immune cells remains unknown. Finally, animal experiments are needed to further elucidate the in vivo role of NPM3. We anticipate that further studies will shed light on the action pattern of NPM3 in the LUAD tumor microenvironment.

## Conclusion

To conclude, our study revealed that NPM3 expression is significantly elevated in LUAD tissues and contributes to the growth and migration of LUAD cells in vitro. We demonstrated that NPM3 may be regulate the expression of cell cycle pathway-related genes, as well as impact immune-related genes and immune cell infiltration, to achieve this function. Our findings provide valuable insights for further investigation into the role of NPM3 in LUAD. Moreover, targeting NPM3 may present a novel therapeutic strategy for LUAD treatment.

## Supplementary Information

**Additional file 1: Supplementary Figure S1.** *NPM3*expression in different clinicopathological parameters of TCGA-LUAD.

**Additional file 2: Supplementary Figure S2.** *NPM3*mutation in cancer.

**Additional file 3: Supplementary Figure S3.** Single-cell clustering plots and *NPM3* expression in GSE131907, GSE143423 andGSE146100 datasets.

**Additional file 4: Supplementary Table S1.** Primer sequences.

**Additional file 5: Supplementary Table S2.** A protein-protein interactionnetwork parameter based on NPM3.

**Additional file 6: Supplementary Table S3.** The genes positively associated with *NPM3* expression.

**Additional file 7: Supplementary Table S4.** The KEGG enrichment pathway of genes positivelyassociated with *NPM3* expression.

**Additional file 8: Supplementary Table S5.** The genes negatively associated with *NPM3* expression.

**Additional file 9: Supplementary Table S6.** The KEGG enrichment pathway of genes negativelyassociated with *NPM3* expression.

## Data Availability

The data used to support the findings of this study are available from the TCGA, GTEx, HPA, and UALCAN database.

## Abbreviations

LUAD: Lung adenocarcinoma
NSCLC: Non-small cell lung cancer
NPM3: Nucleophosmin3
ES: Embryonic stem
TP2: Transition protein 2
sEV-AT: Small extracellular vesicles
OS: Overall Survival
DFS: Disease Free Survival
PFI: Progression Free Interval
DFI: Disease Free Interval
siRNA: Small interfering RNA
Mono/Macro: Monocytes/Macrophages
DC: Dendritic cells
NAFLD: Nonalcoholic fatty liver disease
CAMs: Cell adhesion molecules
TCGA: The Cancer Genome Atlas
GTEx: Genotype-Tissue Expression
HPA: Human Protein Atlas
UCSC: UNIVERSITY OF CALIFORNIA SANTA CRUZ
cBioPortal: CBio Cancer Genomics Portal
UALCAN: The University of ALabama at Birmingham CANcer data analysis Portal
IHC: Immunohistochemistry
qPCR: Real-time quantitative PCR
scRNA-seq: Single-cell RNA sequencing
MHC: Major histocompatibility complex
CTL: Cytotoxic T lymphocytes
IFNγ: Interferon-γ
TNFα: Tumor necrosis factor-α
