# Supplementary figures and images for: NPM3 as a novel oncogenic factor and poor prognostic marker contributes to cell proliferation and migration in lung adenocarcinoma
Source: Hereditas. 2023 May 31;160:27. doi: 10.1186/s41065-023-00289-6 (PMC10230701; doi:10.1186/s41065-023-00289-6)

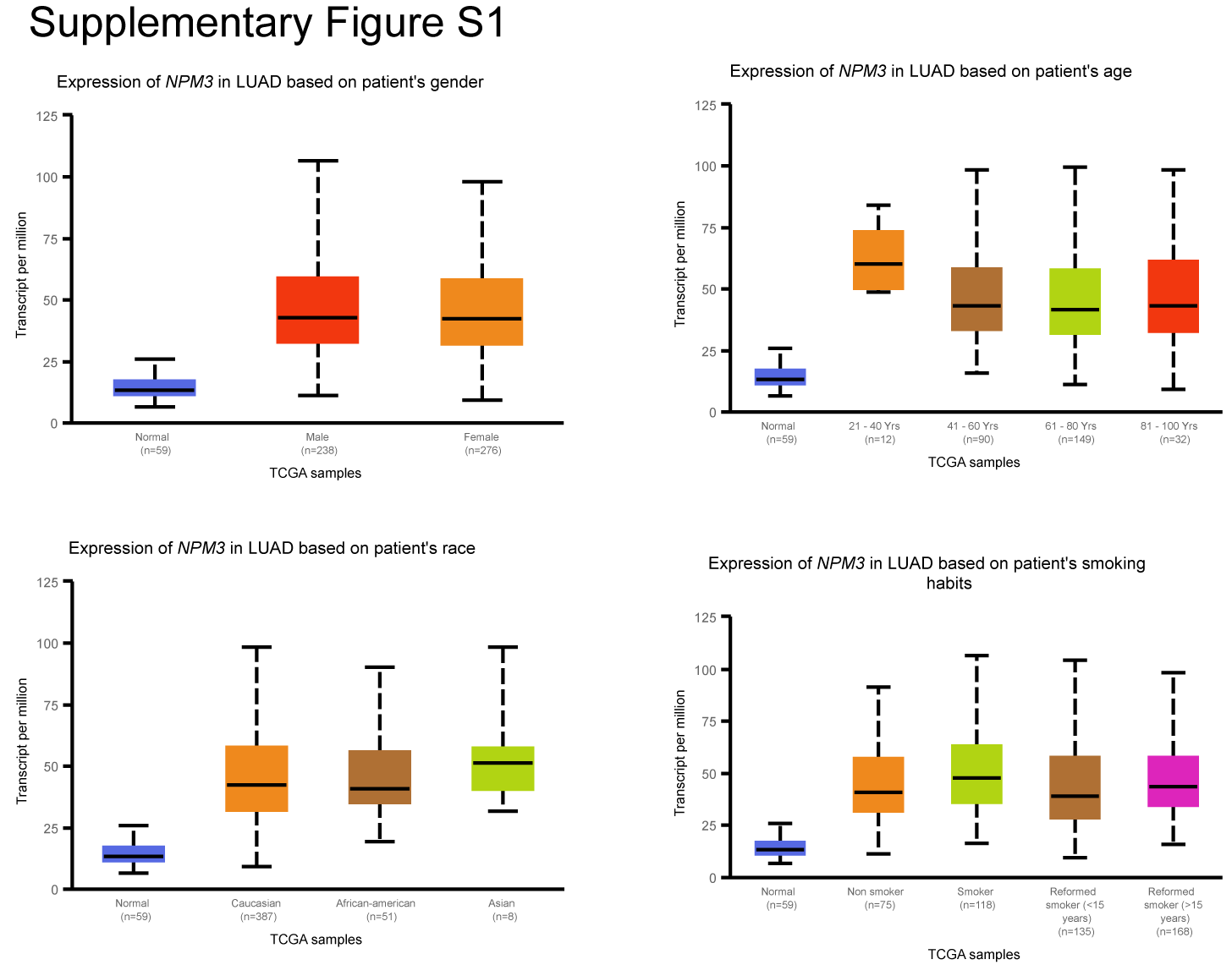

Supplement: Supplementary file 1 — Additional file 1: Supplementary Figure S1. NPM3expression in different clinicopathological parameters of TCGA-LUAD. [file 41065_2023_289_MOESM1_ESM.tif]

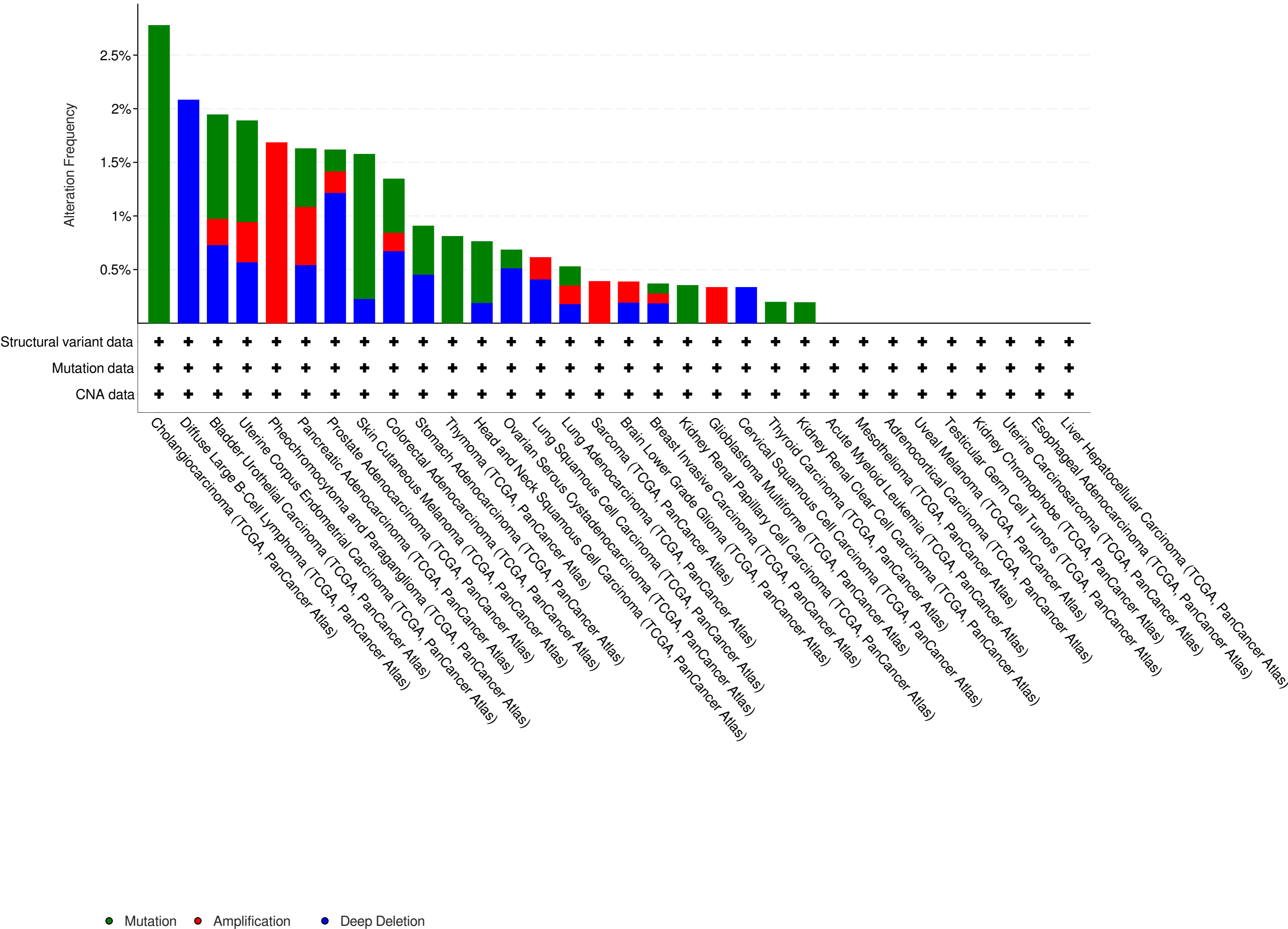

Supplement: Supplementary file 2 — Additional file 2: Supplementary Figure S2. NPM3mutation in cancer. [file 41065_2023_289_MOESM2_ESM.pdf]

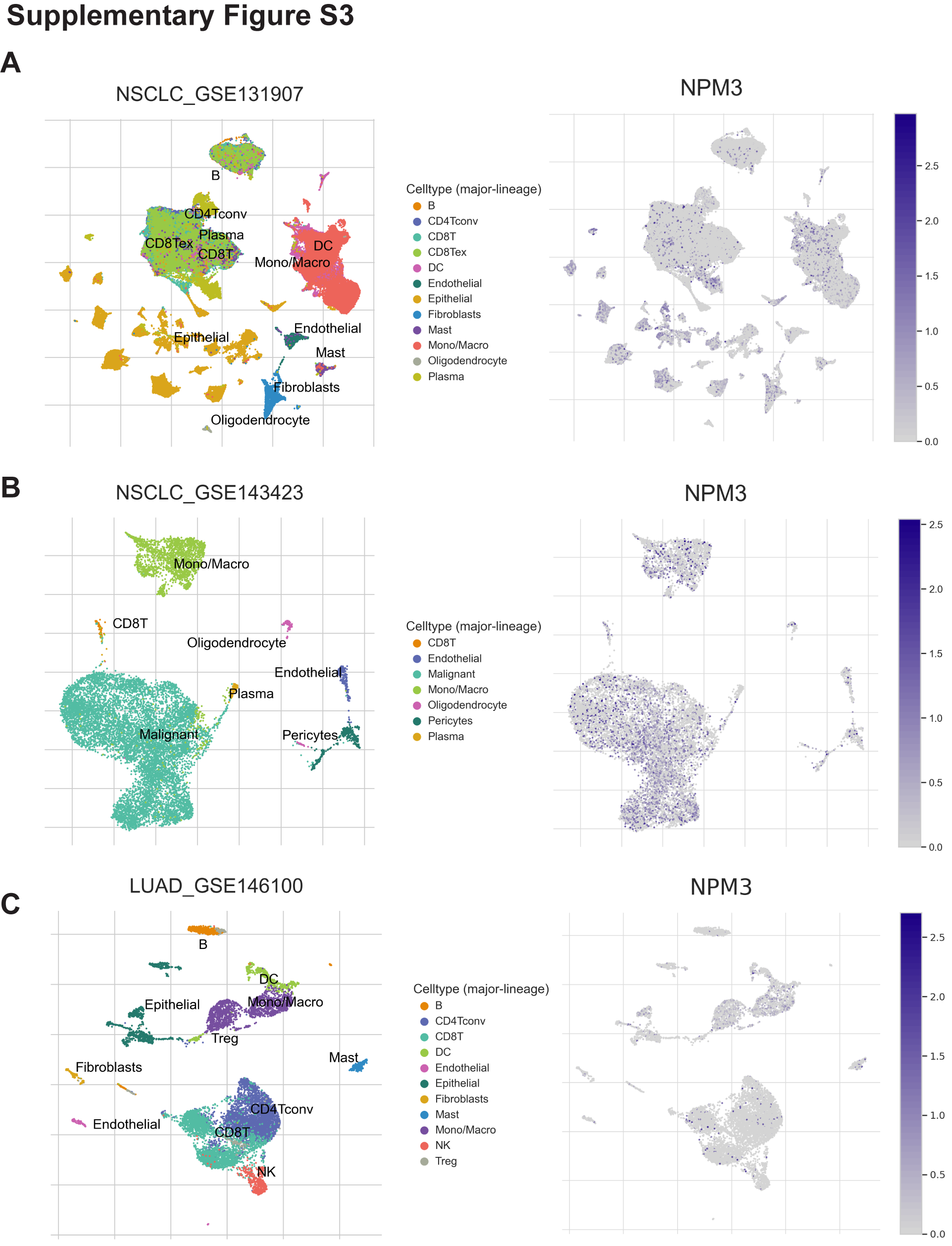

Supplement: Supplementary file 3 — Additional file 3: Supplementary Figure S3. Single-cell clustering plots and NPM3 expression in GSE131907, GSE143423 andGSE146100 datasets. [file 41065_2023_289_MOESM3_ESM.tif]
